# Workplace aggression, wellbeing, and job satisfaction: The specificity in border police organizations

**DOI:** 10.3389/fpsyg.2022.1004153

**Published:** 2022-10-13

**Authors:** Concha Antón, Merlin Patricia Grueso-Hinestroza, Juan C. Espinosa, Mirela Turc

**Affiliations:** ^1^Department of Social Psychology and Anthropology, Universidad de Salamanca, Salamanca, Spain; ^2^School of Management and Business, Universidad del Rosario, Bogotá, Colombia; ^3^General Inspectorate of Romanian Border Police, Psychology Office, Bucarest, Romania

**Keywords:** workplace aggression, job satisfaction, wellbeing at work, police organization, gender

## Abstract

In male-dominated work contexts, the challenges faced by women and their impact on wellbeing and work attitudes have been documented, most extensively in other than police organizations. This study was conducted as a cross-sectional quantitative descriptive correlational predictive study to validate a model of relationships among workplace aggression, job satisfaction, and wellbeing at work with a sample of 1,066 female and male officers from the Romanian Border Police. The results obtained in the study showed that no differential gender effects were found, although women reported higher levels of workplace aggression than men (1.61 vs. 1.52; *F* = 4.20, *p* = 0.04). Also, workplace aggression significantly and negatively predicted job satisfaction (*R*^2^ = 0.27) and wellbeing at work (*R*^2^ = 0.31). In conclusion, although this research is an exploratory approach to the study of workplace aggression in the Romanian police organization, it can generate interventions that would lead to the reduction of undesirable behaviors such as verbal aggression, malicious jokes, discrimination, perception of inequalities, gossip, and defamatory words. In the future lines of research, different sources and conditions of victims and witnesses can be considered the. We also studied the limitations of the study and the future lines of research.

## Introduction

In Europe, policing involves a series of practices aimed at protecting the free exercise of rights and ensuring security and justice. Paradoxically, evidence suggests that law enforcement officers are particularly susceptible to workplace aggression (Segurado et al., 2008; Yu and Lee, 2020; Chitra and Karunanidhi, 2021).

Researchers’ interest in the study of workplace aggression is based on its high prevalence in organizations and the apparently limited capacity of norms and protocols to prevent it (Ragins and Cornwell, 2001). Despite certain limitations linked to methodological aspects and the wide range of figures offered (Barling et al., 2009), evidence suggests that its prevalence is higher in public organizations. In addition to the high tolerance of workplace aggression being common in other types of organizations, the reasons for this include low mobility of employees, difficulty in escaping from one’s reputation when changing positions within the same organization, and the bureaucratic nature of the organization (Zapf et al., 2003). Other factors include stressful and competitive work environments (Coyne et al., 2003; Stanton et al., 2021) where there are high-power differentials in hierarchies and an authoritarian leadership style (Mikkelsen and Einarsen, 2001), the requirement for high cooperation (Zapf et al., 2003), work overload (Quine, 2001), perceived uncontrollability (Zapf et al., 2003), and role conflict and ambiguity (Quine, 2001; Jennifer et al., 2003; Agervold and Mikkelsen, 2004; Skogstad et al., 2007).

Police organizations, particularly border police, are public service entities rated as highly stressful with a high level of daily (Siu et al., 2015; Wolf, 2017; Demou et al., 2020; Lentz et al., 2020) and uncontrollable risk (Cartwright and Roach, 2021; Chitra and Karunanidhi, 2021; Jackman et al., 2020; Papazoglou et al., 2020b), which face numerous situations, such as humanitarian crises at borders, that have the capacity to generate conflict and role ambiguity for their members (Antón, 2009; Acquadro Maran et al., 2020; Queirós et al., 2020). In these organizations, high cooperation is required (Rexeis, 2017), there is a strong hierarchy, and often an authoritarian leadership style (Piotrowski et al., 2021). All of these characteristics put the work environment at a potential risk of experiencing workplace aggression (Segurado et al., 2008; Yu and Lee, 2020; Chitra and Karunanidhi, 2021). However, no specific study has been conducted on border policing.

According to empirical evidence, not all people are equally likely to be victimized by workplace aggression. Gender, age, seniority, position, ethnic or racial group, and affective-sexual orientation were the variables analyzed to identify the potential victims (Aquino and Thau, 2009; Stanton et al., 2021). In the case of police and other male-dominated organizations, the most studied group, especially in relation to sexual harassment, has been women (García, 2021). In general, it has been argued in academia that women are more likely to suffer workplace aggression (Chitra and Karunanidhi, 2021), although in practice, there seems to be no awareness of this reality (Antón et al., 2021).

The presence of women in police organizations has been described as tokenistic due to their under-representation in the workforce, especially in operational positions and at high hierarchical levels (García, 2021). This under-representation has been associated in the literature with innumerable undesirable effects on their wellbeing such as perception of isolation, loss of identity, low self-esteem, low self-efficacy, stress (Castelhano et al., 2012; Morabito and Shelley, 2018; Chu, et al., 2020) and also low job satisfaction, and lack of affective and continuance commitment (Krimmel and Gromley, 2003; Guilliaume et al., 2011; Stroshine and Brandl, 2011).

Among the affective consequences of exposure to workplace aggression that have received, the most attention in the literature is emotional wellbeing at work (Schat and Kelloway, 2000; LeBlanc and Kelloway, 2002; Nel and Coetzee, 2020; Alfandari et al., 2022) and job satisfaction (Budd et al., 1996; Keashly et al., 1997; Tepper, 2000; Quine, 2001; Vartia and Hyyti, 2002; Lapierre et al., 2005; Duffy et al., 2006; Aquino and Thau, 2009; Rodríguez-Muñoz et al., 2009; Caillier, 2021), probably because of the important organizational and personal consequences associated with both constructs. In sum, the impact of workplace aggression on wellbeing at work and job satisfaction not only has a potential personal cost but also affects performance and society as a whole.

In academic literature, psychological wellbeing is related to desirable aspects such as positive effect, self-esteem, and life satisfaction, while its absence is related to the prevalence of psychological distress such as negative effect, stress, and low life satisfaction (Jackman et al., 2020). On the other hand, job satisfaction, resulting from the emotional response to the events workers experience in organizations, is a variable that is associated with organizational commitment and job performance (Antón, 2009; Kumar, 2021; Nalla et al., 2021; White et al., 2021). Although these topics have some relevance in studies conducted in police organizations, they have been investigated less frequently (Jackman et al., 2020; Papazoglou et al., 2022), and only a few have investigated the impact of workplace aggression on them (Simmons-Beauchamp and Sharpe, 2022), in particular, on women (Bastarache, 2020; García, 2021).

Border police in Europe include among their functions the work performed in border crossing points and navy for border surveillance, migration, border crossing criminality prevention and combat, and operational support and participation in FRONTEX missions. The Romanian Border Police is a relatively young body, the result of a demilitarization process in 2002, which currently operates as a police-type institution with a national scope of action. It is composed of the General Inspectorate of the Border Police, which is the central structure, subordinated to the Ministry of Interior, while the second level is composed of the 5 Territorial Inspectorates (Giurgiu, Timișoara, Oradea, Sighetu Marmației, and Iași) and the Coast Guard. It is responsible for guarding 3,147 km of borders, of which 2,070 km represents the second-longest external border in the EU. At the time of this study, there were approximately 12,000 employees of which 2,600 were women.

According to data provided by EUROSTAT (2022), female police officers have an average representation of 21.28% in European police forces, so the percentage of women in the Romanian Border Police is in the average range. Organized along similar lines to the model of the institutions of the EU states, it has made significant efforts in the field of preventing and combating cross-border criminal phenomena and in the implementation and enforcement of the existing internal and international legislation, so that at the Romanian borders and its specific activities match international standards. Due to their functions and nature, border police forces are highly stressful work environments in which the occurrence of workplace aggression can have an incremental effect with undesirable consequences, particularly in women. In view of all the above reasons, the aim of this research is to analyze the impact of exposure to workplace aggression on the job satisfaction and wellbeing of female and male border police officers, and to analyze whether this effect occurs differentially.

## Theoretical framework

### Workplace aggression

Psychological harassment, bullying, workplace trauma, scapegoating, work abuse, victimization, petty tyranny, emotional abuse, oppression, subrogation, abusive behavior, antisocial behavior at work, psychological terror, moral harassment, sexual harassment, psychological violence in the workplace, psychological harassment, mobbing, bossing, discrimination, incivility, and workplace aggression are some of the names used to describe situations of violence in the workplace (De Elena, 2005; Neall and Tuckey, 2014). In addition, scholarship has narrowed down the forms of victimization, distinguishing the sexual ones from the rest, as well as according to the agent, the intensity of the behaviors, and their persistence over time. Other authors have attempted to develop broad definitions of the construct capable of encompassing a wide variety of behaviors whose ultimate aim is to cause harm to the work environment.

Schat and Kelloway (2000, p. 191) define workplace aggression as “behavior by an individual or individuals within or outside an organization that is intended to physically or psychologically harm a worker or workers and occurs in a work-related context.” Some authors have pointed out that coworkers and managers are the main sources (Al-Shamaileh et al., 2022). In the framework of this research, workplace aggression is understood as an over-arching construct that encompasses all behaviors intended to cause psychological but also physical harm, regardless of whether these are direct or indirect, from the agent causing the harm (superior, co-worker, subordinate, citizen) including sexual harassment and incivility at work. This approach is consistent with research that highlights similar behaviors of different forms of workplace violence (Buchanan and Fitzgerald, 2008), including indirect aggression (Ragins and Cornwell, 2001; Dionisi and Barling, 2018), and that “facilitates the inclusion of different bodies of literature that cover essentially the same antecedents and consequences of negative workplace interactions” (Neall and Tuckey, 2014, p. 226).

Aquino and Thau (2009) consider that workplace victimization occurs “when an employee’s wellbeing is harmed by an act of aggression perpetrated by one or more members of the organization” (p. 718). In this context, the authors related wellbeing to the satisfaction of psychological and physiological needs, including a sense of belonging, the feeling of worthiness, the belief that one has the ability to predict and cognitively control one’s environment, and being able to trust others.

A significant amount of research on workplace aggression has examined the differences between men and women with respect to the exposure and perceived severity of such behaviors. Although a considerable number of cases have found a higher prevalence among women (Björkqvist et al.,1994; Aquino and Bradfield 2000; Cortina et al., 2001; Salin, 2001, 2003; Tehrani, 2004; Escartin et al., 2013), particularly in cases of intersectional harassment (Rabelo and Cortina, 2014), no consistent pattern of relationship can be affirmed in the research, as more than a few studies have found it to be an irrelevant variable (Einarsen and Skogstad, 1996; Leymann, 1996; Vartia and Hyyti, 2002; Hansen et al., 2006) or prevalent in men (Jennifer et al., 2003).

This disparity in results appears to be related to the role played by other variables in men’s and women’s perceptions such as the type of behaviors studied, the gender and status of the harasser, the gender ideology of the observer, organizational tolerance of harassment, and the age and seniority of those being harassed (Sigal and Jacobsen, 1999; McCabe and Hardman, 2005; Bowling and Beehr, 2006). In a meta-analysis, Rotundo et al. (2001) found that women perceived a wider range of socio-sexual behaviors as harassment than men, although the size of the difference was not large. The meta-analysis found significant differences in perceptions between women and men regarding hostility in the work environment, derogatory attitudes towards women, and pressure in dating or physical sexual contact, but none in the case of sexual advances or sexual coercion. Other analyses, such as that by Stockdale et al. (2004), find that people with a sexist patriarchal worldview are particularly insensitive to the same-sex rejection-based harassment of men, a form of harassment that is particularly prevalent in men.

On the other hand, it is important to distinguish between the recognition of the existence of violent behaviors in the work environment and self-categorization as bullied or discriminated against. The attributional model of bullying by Samnani et al. (2013) argues that exposure to negative behaviors will hardly be perceived as bullying when there is high consensus (they are very common), high consistency (they are spread over time and normalized), or low distinctiveness (they are directed at the whole group). This and other studies (e.g., Tuckey et al., 2009) highlight the importance of context in the occurrence and perception of different forms of workplace aggression.

In police contexts, several studies on workplace aggression have focused on women as targets of sexual harassment (Shadmi, 1993; Martin, 1996). Although the inclusion of women in European policing began with their incorporation into ancillary, women’s, and childcare work, policing models in Europe today follow an inclusive strategy. This strategy, promoted by laws prohibiting gender discrimination in the workplace, encounters, different forms of resistance that imply the real maintenance of inequalities between men and women according to Brown (1997), and which include, among others, discrediting, the spread of rumors, and sexual harassment.

Research on police officers on all continents reveals the persistence of a police culture that emphasizes the values associated with masculinity and questions the role of women in the police (Antón et al., 2021). In relation to workplace aggression, this culture not only favors the existence of violence against policewomen but also supports the organization’s tolerance of such violence (Simmons-Beauchamp and Sharpe, 2022).

The rejection of the presence of women within the police has been related to the threat to the maintenance of hegemonic masculinity, which would be challenged along with the acceptance of unnecessary use of force and myths about policing and masculinity (Hunt, 1984; Herbert, 2001; Messerschmidt, 2016). This rejection translates to different obstructive behaviors. As García, (2021) argues, “Nothing makes resistance to women in policing more obvious than gender discrimination, sexual harassment, and assault in policing” (p. 119). Furthermore, Wade and Brittan-Powell (2001) found that not only men who endorse an ideology of traditional masculinity, but also men who rely on a male reference group for their professional self-concept, are anti-gender equitable and prone to sexual harassment, which is relevant in police organizations.

On the other hand, ironic as may seem from the analysis of the content of police work and the proven effectiveness of policewomen (Herbert, 2001), the wrong gender approach permeates police culture and reaches institutional practices, including selection, promotion, evaluation processes, decision-making, and the organization of work itself (Natarajan, 2008; García, 2021). In a qualitative study of 28 European police officers, Antón et al. (2021) found widespread perception of gender discrimination in institutional practices among policewomen.

Recent research continues to highlight the prevalence of certain forms of workplace aggression towards women in police settings. Steinþórsdóttir and Pétursdóttir (2018), for example, examine different forms of violence in the Icelandic police force. In their study, women were 10 times more likely to experience sexual harassment than men by their male supervisors, colleagues, subordinates, and the male public. Significantly, the figures show that harassment more often comes from within rather than from outside, which reinforces the idea of resistance to women joining police organizations. Men however find that they are more bullied than women (Steinþórsdóttir and Pétursdóttir, 2018).

On the other hand, a notable finding in several research studies is that female police officers tend to either engage in neutralizing harassing behavior or accept it as a way to fit into organization (García, 2017; Brown et al., 2020).

### Job satisfaction and workplace aggression

Lambert et al. (2016) defined job satisfaction as “an effective/emotional response by an employee concerning his/her particular job and whether the employee likes the job’ (p. 23). It is a positive emotional state resulting from an individual’s evaluation of their work experiences (Locke, 1976), which implies that job satisfaction derives from employees’ judgment of the characteristics of the job and work environment.

Studies of the antecedents of job satisfaction have been based on three broad dimensions: individual, task-related, and environment-related characteristics (Johnson, 2012; White et al., 2021). Regarding individual characteristics, evidence has led to the conclusion that variables such as race, gender, or education have a weak or inconsistent relationship with job satisfaction (White et al., 2021). On the other hand, studies analyzing the relationship between job satisfaction and task characteristics have shown statistically stronger relationships, especially in circumstances where workers consider that their work makes a relevant social contribution (White et al., 2021). Finally, studies analyzing the characteristics of the organizational environment and its relationship with job satisfaction show that job satisfaction is best explained by variables related to organizational support and coworker relationships (Zeng et al., 2020; White et al., 2021).

Although there is a widely accepted assumption that workplace aggression experiences have a negative impact on job satisfaction (Ertureten et al., 2013), some research findings suggest that the relationship between the two variables is not simple. Some authors stress the need to contextualize experiences to understand their meaning and how they affect satisfaction. For example, in a study analyzing a sample of local police officers, Segurado et al. (2008) found that discriminatory practices are widespread and supported by a corporate culture that values power, hierarchy, and control as guiding principles of internal functioning, so that they are assumed to be a part of the nature of police work. In other work environments, greater acceptance of inequalities has been found (Loh et al., 2010). Some national cultures, masculine organizational cultures (Power et al., 2013), and cultures that are characterized by a low ‘human orientation’ may be more tolerant or even accepting of discriminatory behavior so that it does not have a negative effect on job satisfaction Giorgi et al. (2015). These arguments align with the hypotheses proposed from the attributional model of bullying by Samnani et al. (2013).

In their meta-analysis, Lapierre et al. (2005) found that the impact of workplace aggression on satisfaction was weaker for men than for women. Lutgen-Sandvick et al. (2007) found that witnesses of workplace bullying had lower job satisfaction, although higher than those who were directly bullied. Giorgi et al. (2015) pointed out that satisfaction is more a result of people’s perceptions and self-labeling than of actual exposure to violent behavior in the work environment.

### Wellbeing at work and workplace aggression

Wellbeing is conceived as a multifaceted construct that can be viewed from physical, emotional, psychological, and mental perspectives (Gerhardt et al., 2021). Specifically, it describes how well individuals feel in life, including the social, health, and material aspects (Diener et al., 2018). Thus, a low level of wellbeing is a strong predictor of negative psychological outcomes, such as sleep disorders, depression, anxiety, fatigue, burnout, and depression (Onyishi et al., 2021). Similarly, empirical evidence indicates that low levels of wellbeing are related to behavioral problems, such as suicidal ideation and alcohol abuse, and physiological symptoms, such as headaches, decreased immune function, musculoskeletal pain, and cardiovascular disease (Onyishi et al., 2021). Finally, at the organizational level, low levels of wellbeing are related to absenteeism, inefficiency, problem-solving ability, creativity, work engagement, and productivity (Onyishi et al., 2021).

Workplaces play a crucial role in workers’ psychological wellbeing. (Jackman et al., 2020). Research has consistently found a direct and negative effect of workplace aggression experiences on psychological wellbeing at work (Buchanan and Fitzgerald, 2008), which has been explained by the excessive physical and psychological arousal involved in dealing with them. Although some authors have concluded that it is the subjective experience of victimization that has a negative impact on wellbeing (Nielsen and Einarsen, 2012), in many other cases, mere exposure seems to be sufficient to generate a decrease in workers’ wellbeing (Dehue et al., 2012; Giorgi et al., 2015). Hewett et al. (2018) analyzed the potential moderating effect that self-labelling would have on the relationship between exposure to bullying behaviors and wellbeing, finding that the effect of self-labelling was very small, although it was a determinant in the coping strategies developed by workers. Research suggests that workers’ psychological wellbeing decreases when they witness the bullying of another person, that is, when victimization is indirect (Hoel et al., 2004; Dionisi and Barling, 2018; Sprigg et al., 2019).

The relationship between workplace aggression and police wellbeing has been little or only tangentially studied. Ellrich (2016) found that burnout increases patrol cops’ risk to violent victimization but also favors the development of positive attitudes toward the use of violence. This study focuses exclusively on exposure to workplace aggression developed by citizens against police officers. Police officers interviewed by Milliard (2020) included workplace bullying as a source of stress. Recently, Bastarache (2020) reported that discriminatory and sexual harassment deteriorate the occupational health of Canadian police officers. Focusing on the moral damage caused by involvement in events that deeply transgress one’s moral values and expectations, such as workplace aggression, either as a perpetrator or passive witness, Papazoglou et al. (2020a) found that this has an important impact on the development of post-traumatic stress disorder in police officers.

Bourbonnais et al. (2007) and Ricciardelli et al. (2020) found that in Canadian correctional officers, intimidation and psychological harassment at work increased the likelihood of psychological distress, especially in women. In another environment close to police organizations in terms of culture and gender imbalance, Fitzgerald et al. (1999) conducted a study with 28,000 military personnel in which they tested an integrated model of sexual harassment. Their results showed that the processes of sexual harassment are the same for men and women in the military, although the impact of sexual harassment on work satisfaction and psychological wellbeing was more pronounced for women than for men. In contrast, Parker and Griffin (2002) found that sexual harassment follows different patterns for male and female police officers, with no impact on men’s distress. De Haas et al. (2009) in research with police officers who had experienced sexual harassment report a similar impact on the mental and physical health of men and women, although female police officers were more frequently harassed and more concerned about this issue. The possibility of a differential impact of harassment between male and female police officers has been rarely studied, with inconclusive empirical evidence, and the authors stress the need for further research in this area (De Haas et al., 2009; Krakauer et al., 2020).

## Research aim and hypotheses

This study analyzes the relationships between workplace aggression and the wellbeing and job satisfaction of border guards. According to the literature review, it is possible that there are differences between male and female border guards; therefore, we also explore this possibility.

**Figure 1:**
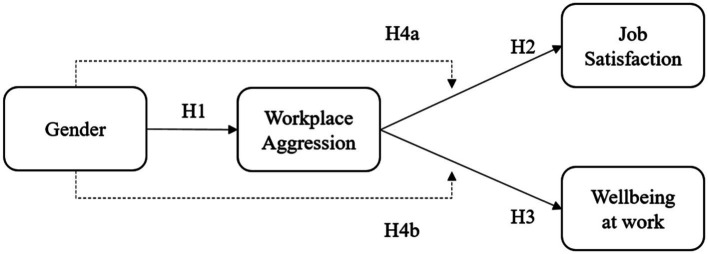
Hypothesized model.

As a result, in the present study, four hypotheses were structured, in which (1) female police officers will report more experiences of workplace aggression than their male counterparts (H1), (2) workplace aggression is predictive of participants’ job satisfaction (H2) and wellbeing at work (H3); the relationship between workplace aggression and psychosocial outcomes varies according to gender (H4) 4a (workplace aggression and job satisfaction) and 4b (workplace aggression and wellbeing at work; see Figure 1).

## Materials and methods

### Design

This study is a descriptive correlational research with predictive purposes in which a multi-group analysis of the partial least squares (PLS) was carried out.

### Participants and procedure

This study involved 1,100 officers from the Romanian Border Police. Regarding the sample strategy we used a stratified random sampling technique. The layers were the territorial border guard structures from every district located on the Romanian borders, all the sectorial structures and the border crossing points in the airports. At least two respondents were randomly chosen from each specialized working area (BPC, LBS, N, MC, and OS) and every organizational structure. The sample was reduced to 1,066 (96.91%) because of the exclusion of 34 records due to atypical scores on the variables of analysis. Of the final sample, 16.4% were female. Descriptive statistics regarding the measures are in Table 1.

**Table 1:** Demographic profile of participants of the study (*N* = 1,066).

Variables	Categories	Freq.(%)
Age	18–25 years old	117 (11.0)
	26–35 years old	295 (27.7)
	36–45 years old	459 (43.1)
	46–60 years old	194 (18.2)
Gender	Male	888 (83.3)
	Female	174 (16.3)
Tenure	0–5 years	280 (26.3)
	5–10 years	81 (7.6)
	10–25 years	619 (58.1)
	25 or more	86 (8.1)
Function	Management	95 (8.9)
	Coordination	151 (14.2)
	Execution	817 (76.6)

Own computation using SPSS.

According to the report Women, Business, and the Law of the International Bank for Reconstruction and Development (2020), Romania, the country to which the sample belongs, is among the 86 countries out of the 196 analyzed that achieve the highest score in the labor index for the existence of anti-discrimination and anti-harassment protection measures in the workplace. These rules have been transposed into the internal rules and regulations of the police force, of which 22.3% are women (EUROSTAT, 2022).

### Instruments

The questionnaire contained sociodemographic variables related to age, gender, seniority and function (hierarchical position), as illustrated in Table 1. In addition, the questionnaire analyzed three constructs: workplace aggression, job satisfaction, and wellbeing at work (see Table 2). Workplace Aggression: This includes six items on a four-point Likert scale (never = 1, rarely = 2, often = 3, and always = 4) and indicates the degree to which individuals are exposed to harassment and discrimination, both personally and as a witness (e.g., Are your workers exposed to gossip, slanderous, and defamatory words in your team?).

**Table 2:** Descriptive statistic, and reliability of measure (*N* = 1,066).

Variables	Min.	Max.	Median	Mean	SD	Cronbach’s alpha	CR	AVE
Workplace aggression	1.00	4.00	1.50	1.54	0.52	0.88	0.91	0.63
Job satisfaction	1.00	4.00	3.25	3.38	0.49	0.83	0.89	0.66
Wellbeing at work	1.67	4.00	3.83	3.60	0.43	0.88	0.90	0.61

Composite reliability (CR) and average variance extracted (AVE).

Composite reliability (CR) and average variance extracted (AVE).

Own computation using PLS.

Job satisfaction: This includes four items in which participants rate their job satisfaction using four options (to a very small extent = 1; to a small extent = 2; largely = 3; to a very large extent = 4). An example of these items is: Does work give you professional satisfaction?

Finally, wellbeing at work was analyzed, which included six items in which participants rated their wellbeing in relation to work in four options (to a very small extent = 1, to a small extent = 2, Largely = 3, to a very large extent = 4). An example of these items is: Do you feel tense at work? (reverted). Descriptive statistics regarding the measures are in Table 2.

### Ethics statement

In accordance with local legislation and institutional requirements, ethical review and approval were not required for this study on human participants. Written informed consent for participation (regarding the purpose of the study, anonymity of data, and use of data) was obtained prior to the completion of the questionnaires.

### Procedure

The questionnaire was administered by 15 psychologists to 1100 border guards from Romanian Border Police. Once informed consent was obtained, participants completed the questionnaires, which were processed using SPSS statistical software (IBM Corp, 2020) to calculate descriptive statistics, and SmartPLS (Ringle et al., 2015) to validate the relationship model between the study variables.

## Results

### Sociodemographics and descriptive results

First, the sociodemographic characteristics of the study participants were identified. Approximately half (43.1%) of the participants were between 36 and 45 years of age, mostly male (83.3%), with extensive experience in the position (58.1% with seniority between 10 and 25 years), and occupying operational level positions (76.6% in execution). See Table 1.

To analyze the properties of the measurement scales used, Cronbach’s alpha, composite reliability, and average variance extracted were estimated to obtain a reliable measure (Hair et al., 2017). Similarly, standardized root mean square and normed fit indices were estimated for the adjustment index of the model (Henseler et al., 2016). Regarding the reliability of the measures, it was found that the three questionnaires presented adequate levels of internal consistency with values above 0.70 Cronbach’s, composite reliability (CR), and average variance extracted (AVE) above 0.50 (Table 2). Finally, the quality indicators for the analyzed model were SRMR = 0.06 and NFI = 0.90.

Likewise, the results of the study showed that in the sample studied, wellbeing was the variable with the highest score (3.60), followed by job satisfaction (3.38). Finally, workplace aggression was the variable with the lowest score (1.54), as described in Table 2.

### Hypothesis testing

**Figure 2:**
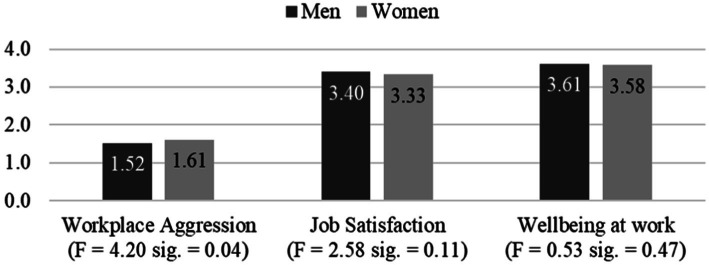
Workplace Aggression, Job Satisfaction, and Wellbeing at Work by gender.

To test Hypothesis 1 regarding gender differences in the experience of workplace aggression between males and females, the mean difference statistic was run. The statistical results obtained indicate that there are no significant differences in the scores obtained by men and women in the Job Satisfaction (*F* = 2.58, *p* = 0.11) and Wellbeing at Work (*F* = 0.53, *p* = 0.47) variables, but there are significant differences in the Workplace Aggression measure (H1; *F* = 4.20, *p* = 0.04), thus H1 was supported (Figure 2).

**Figure 3:**
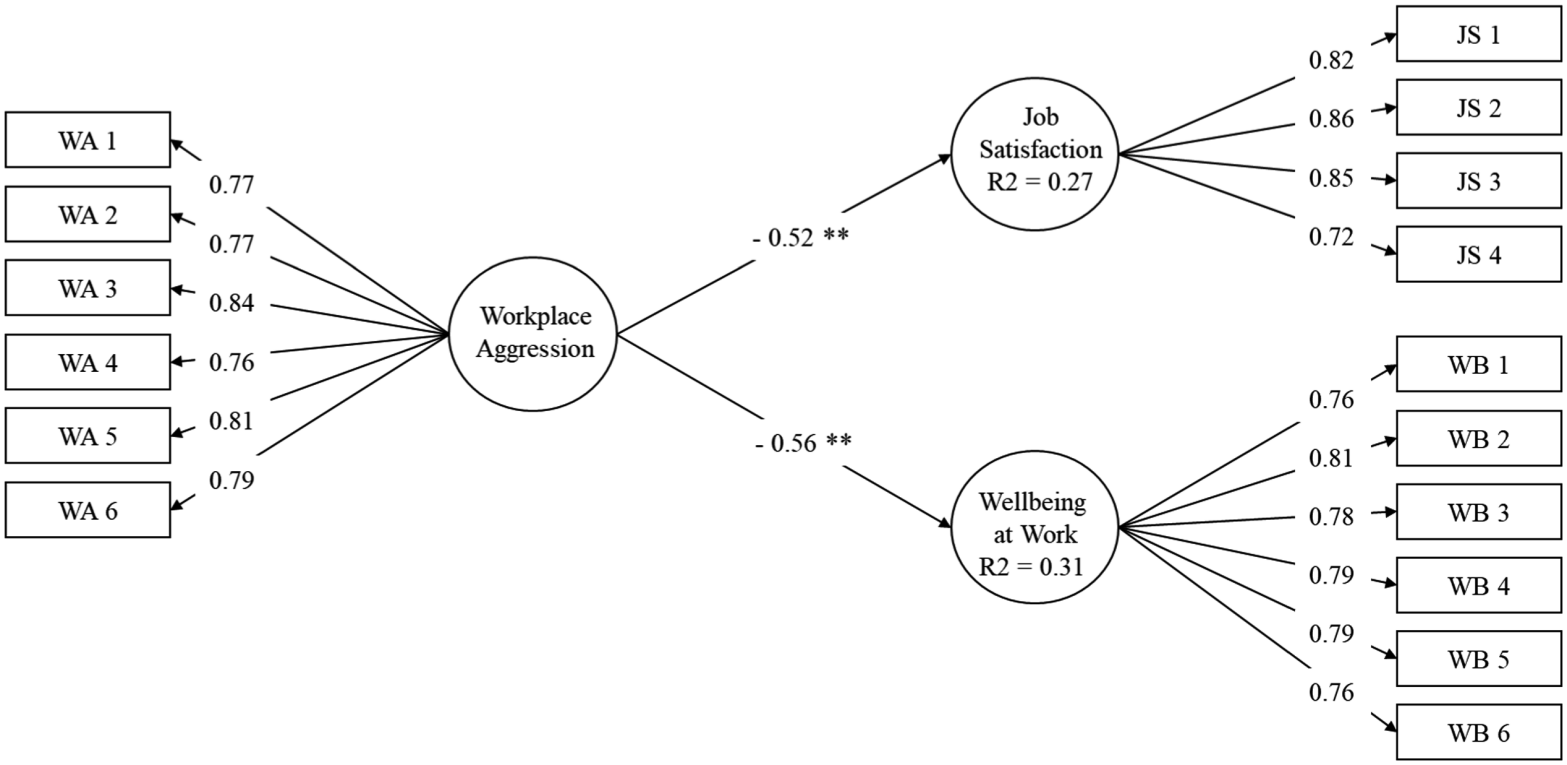
Effects of the Workplace Aggression on Job Satisfaction and Wellbeing at Work.

To validate Hypotheses 2 and 3, a structural equation model (SEM) was used (Figure 3). The results showed that workplace aggression had a significant effect on job satisfaction (H2; *R*^2^ = 0.27) and wellbeing at work (H3; *R*^2^ = 0.31). Thus, H 2 and H3 were supported.

Finally, the moderating effect of gender (H4a and H4b) was examined with a multi-group analysis comparing the effect of workplace aggression on job satisfaction, and wellbeing at work separately for men and women. The results show that there are no significant gender differences in the path coefficient, so the moderating effect of gender is rejected (H4 was not supported). Although in both cases, workplace aggression has a higher negative weight in the case of women (−0.54 and –0.64, respectively) as illustrated in Table 3.

**Table 3:** Multi-group analysis (gender) in Path-coefficient.

	Path-coefficient	Diff. (men vs. female)	*t*-value (men vs. female)	Value of *p* (men vs. female)
WA → JS				
All	–0.52	0.04	0.55	0.58
Men	–0.50			
Female	–0.54			
WA → WB				
All	–0.56	0.10	1.67	0.10
Men	–0.54			
Female	–0.64			

Own computation using SPSS

## Discussion

Workplace aggression has been a research topic of study in different occupational groups, and evidence suggests that law enforcement officers are particularly susceptible to experiencing it (Segurado et al., 2008; Yu and Lee, 2020; Chitra and Karunanidhi, 2021). According to the above four research hypotheses were tested. The results presented in this research show that: (a) female border guards perceive more workplace aggression than male border guards, (b) the likelihood of border guards experiencing job satisfaction decreases when they experience or witness workplace aggression, (c) workplace aggression has a negative impact on wellbeing at work, so Hypotheses H1, H2, and H3 were supported in this research, and (d) contrary to our expectation, we found that the impact of workplace aggression on job satisfaction and wellbeing does not differ between male and female border guards, thus Hypotheses 4a and 4b were not supported.

Regarding exposure and perceived severity of workplace aggression, previous research has shown that there is a prevalence among women (Björkqvist et al.,1994; Aquino and Bradfield 2000; Cortina et al., 2001; Salin, 2001, 2003; Tehrani, 2004; Escartin et al., 2013), although the magnitude of the differences is not large (Rotundo et al., 2001). The results of our research confirm that in the sample of police officers studied, women experienced greater workplace aggression than men. This evidence can be explained in light of the cultural values of police organizations, where the role of women in the police is questioned (Antón et al., 2021) and aggressive behavior toward women is tolerated (Simmons-Beauchamp and Sharpe, 2022).

Several studies have shown that women are more likely to consider certain behaviors as forms of workplace violence (Rotundo et al., 2001). Since in this research workplace aggression has been defined as an overarching construct, which includes the experiences of victimization and its perception as a witness, it is possible to affirm that our results are congruent with the hypothesis of women’s greater awareness of the existence of workplace aggression. Among the reasons that could explain this greater awareness is the history of previous experiences and greater concern about workplace aggression (De Haas et al., 2009), which would not always generate neutralization or denial of such experiences (Brown et al., 2020; García, 2021).

It has been argued that in male-dominated organizations that extol traditional masculinity, such as police organizations, workplace aggression is widespread (Segurado et al., 2008) and accepted as part of the job (Loh et al., 2010; Power et al., 2013); this fact would explain why workplace aggression, in such contexts, would not have an impact on job satisfaction (Giorgi et al., 2015). However, the results showed that border guards participating in our study, regardless of whether they were male or female, developed lower job satisfaction when they perceived and experienced workplace aggression. This result questions whether border guards normalize victimization and discrimination at work. The existence of anti-discrimination and anti-bullying norms in organizations, although not capable of eradicating workplace aggression, seems to influence the organizational cultural values shared by their members (Ragins and Cornwell, 2001).

It is possible, therefore, that the so-called police subculture is now beginning to become a cultural residue, as stated by the police leaders interviewed by Antón et al. (2021) and many of the female police participants in Yu and Lee’s research (2020). It is also possible that attitudes toward workplace aggression in police organizations, as in other settings, mutate toward more ambivalent manifestations, in which discriminatory and violent behaviors are combined, while negative cognitions toward unequal treatment are maintained (Glick and Fiske, 2001; McCabe and Hardman, 2005; Feather and Boeckmann, 2007).

Additionally, previous research has shown a negative statistical relationship between workplace aggression and job satisfaction (Ertureten et al., 2013). In our research, we found that this relationship is explained by police officers’ experiences related to verbal aggression, malicious jokes, discrimination, perception of inequalities, gossip, and defamatory words generating low job satisfaction in the analyzed sample.

On the other hand, this research shows that psychological wellbeing decreases when individuals experience or witness workplace aggression behaviors, results that are in line with previous evidence. For example, in their research Milliard (2020) found that workplace aggression, expressed through bullying, negatively impacts the police officer’s wellbeing. In addition, Bastarache (2020) reported that discriminatory and sexual harassment has a strong negative impact on occupational health in Canadian police officers. Papazoglou et al. (2020a) found that workplace aggression has a strong impact on police officers’ wellbeing.

United Nations Resolution 1,325 on Women, Peace, and Security highlights the contribution of policewomen to the wellbeing of society as a whole and to the maintenance of peace. However, their under-representation in police organizations, discrimination, and harassment within them are indicative of a lack of full integration (Natarajan, 2008; Antón et al., 2021; García, 2021; Simmons-Beauchamp and Sharpe, 2022).

Much of the research conducted in police contexts has been developed in Western countries. There is some discussion about the extent to which these findings can be extrapolated to other cultures, especially the former Soviet block nations in the east of Europe (Yu, 2002). Social, economic and political background has been considered to play an important role in the culture of police organizations (Ganapathy and Cheong, 2016). For example, an international project on police integrity that studied attitudes towards misconduct in police organizations found differences between Eastern and Western European countries (Ekenvall, 2003). Constantinou and Butorac (2019) analyse the presence of police values considered to be Anglo-American police subculture in European countries with totalitarian pasts and find that police subculture is present in all of them. The results of the research conducted with the Romanian Border Police are in line with this position.

Clearly, the results of this study show that the perception of workplace aggression has a significant negative impact on the wellbeing at work experienced by male and female border guards. Although this relationship is stronger for women than for men, the differences are not significant. Some authors have argued that women are more aware of their emotions, and this is reflected in their questionnaire scores (Mankus et al., 2016), but our results do not allow us to confirm this difference.

## Conclusion

In this research, we have used an overarching approach to analyze aggression at workplaces. Although we believe that this is appropriate for the exploratory nature of the research, it prevents us from analyzing the differential impact that different forms of harassment and discrimination may have and the analysis of the concomitants that they may have on border guards.

Based on the results obtained, we can conclude that women in the study sample perceived workplace aggression more intensely than men. Likewise, the results showed that workplace aggression affects psychosocial variables at work, such as job satisfaction and wellbeing, although no differential results were found between men and women.

To reduce the adverse effects of workplace aggression on police officers, police organizations can develop actions aimed at reducing behaviors, such as verbal aggression, malicious jokes, discrimination, perception of inequalities, gossip, and defamatory words, which may be deeply entrenched in organizational cultures with masculinity-centered values. It is also necessary to generate spaces for reflection and debate on how people relate and communicate within organizations. There is sufficient evidence of the implications of civic behavior and good treatment on the wellbeing and satisfaction of workers.

## Limitations and future research

The limitations of this study are as follows: In relation to experiences of workplace aggression, several studies show that the effects vary over time and that this change is especially pronounced in indirect victimization, whose effects decay more rapidly (Hoel et al., 2004; Lutgen-Sandvick et al., 2007; Dionisi and Barling, 2018; Sprigg et al., 2019). The time elapsed since the workplace aggression experience has not been taken into account in this research, and we believe this should be done in the future. On the other hand, we believe that it would be desirable to further explore the effects of workplace aggression on job satisfaction and wellbeing at work, considering the different sources and conditions of victims and witnesses. Furthermore, to avoid possible neutralization effects, it is desirable to use a checklist of situations.

The existence of a police subculture that justifies workplace aggression, particularly in the case of women, has been discussed (Keverline, 2003). It would have been interesting to know the perception of border guards as to whether their organization is inclusive or not. This element could have helped explain the results better. In addition, it is possible that the existence of this subculture determines decisions to report abusive behavior and victims’ resilience. In the future, it should be explored whether pressures to silence exist and what their impact is on reporting behaviors (Keverline, 2003; Collins, 2004; Chaiyavej and Morash, 2009; Yu, 2017; Kutnjak Ivkovic et al., 2018).

The literature on female police officers emphasizes feelings of loneliness and not belonging. O'Reilly et al. (2015) found that ostracism can have even more pernicious effects than harassment, so a future line of research could include ostracism and its relationships, if any, with workplace aggression and other variables.

Finally, this study is correlational and quantitative. We believe that subsequent quantitative studies designed to increase knowledge about how female and male border guards perceive workplace aggression and its impacts on wellbeing and satisfaction should be developed in the future.

## Data availability statement

The datasets presented in this article are not readily available because there is no authorization from the organization from which the data were obtained to be shared outside the research project in which they were generated. Requests to access the datasets should be directed to canton@usal.es.

## Ethics statement

Ethical review and approval was not required for the study on human participants in accordance with the local legislation and institutional requirements. The patients/participants provided their written informed consent to participate in this study.

## Author contributions

All authors listed have made a substantial, direct, and intellectual contribution to the work and approved it for publication.

## Funding

This work was supported by Erasmus+ 2019-1- RO01- KA202-063815.

## Conflict of interest

The authors declare that the research was conducted in the absence of any commercial or financial relationships that could be construed as a potential conflict of interest.

## Publisher’s note

All claims expressed in this article are solely those of the authors and do not necessarily represent those of their affiliated organizations, or those of the publisher, the editors and the reviewers. Any product that may be evaluated in this article, or claim that may be made by its manufacturer, is not guaranteed or endorsed by the publisher.
